# Abdominal War Wounds

**Published:** 1918-05

**Authors:** 


**Abdominal War Wounds.** Miginiac, Le Prog. Med., Nov. 10, 1917, reports 64 penetrating and 36 non-penetrating wounds. 10 of the former were treated by Murphy's operation, all died. In 4, operation was deliberately avoided. 3 died, one, with wound of the bladder recovered. 14 inoperable cases all died. 35 laparotomies were performed: 8 with penetration but no visceral lesion, 5 deaths and 3 cures; 22 with lesion of a single viscus, with 15 deaths and 5 cures (sic) ; 5 cases involving various viscera, all dead. Total mortality, 74%. The highest mortality occurred in penetrations without visceral wound.

Quenu, ibid., Nov. 3, 1917, reports 34 penetrating wounds. In 13, no operation was performed, 4 deaths and 9 cures, 4 being considered inoperable. 1 case was operated by the Murphy method and died. Of 20 laparotomies, 11 died.

